# Cyclic Adenosine Monophosphate: A Central Player in Gamete Development and Fertilization, and Possible Target for Infertility Therapies

**DOI:** 10.3390/ijms232315068

**Published:** 2022-12-01

**Authors:** Jan Tesarik, Raquel Mendoza-Tesarik

**Affiliations:** MARGen Clinic, Camino de Ronda 2, 18006 Granada, Spain

**Keywords:** cyclic adenosine monophosphate, spermatogenesis, oogenesis, fertilization, male infertility, female infertility, infertility treatment

## Abstract

Human infertility, of both male and female origin, is often caused by the deficient response of the testis and the ovary to hormonal stimuli that govern sperm and oocyte development and fertilization. The effects of hormones and other extracellular ligands involved in these events are often mediated by G-protein-coupled receptors that employ cyclic adenosine monophosphate (cAMP) as the principal second messenger transducing the receptor-generated signal to downstream elements. This opinion article summarizes the actions of cAMP in sperm and oocyte development and fertilization, leading to therapeutic actions targeting cAMP metabolism to alleviate human male and female infertility.

## 1. Introduction

Despite the application of new diagnostic and therapeutic methods to deal with human infertility, there has still been a continuous increase in infertility cases over the last 10 years [1]. Cyclic adenosine monophosphate (cAMP) is a second messenger involved in most of the essential molecular events responsible for the development of both male and female gametes. In general, the central role of cAMP in intracellular signal transduction pathways makes it a suitable target for therapies of many human diseases affecting different organs and tissues. However, this article only focuses on infertility therapies. In this short opinion article, we address the mechanism of cAMP action in general as well as its roles in gamete development and fertilization.

## 2. cAMP Function

After its discovery by Dr. Earl W. Sutherland in 1958, there has been accumulating evidence showing that this small hydrophilic molecule is involved in many physiological processes in a variety of cells and tissues [2]. Principally, cAMP is a second messenger, synthesized from adenosine triphosphate (ATP) through cyclization by adenylyl cyclase and eliminated through hydrolysis by cAMP phosphodiesterase. Both the synthesis and elimination of cAMP within cells are regulated by many types of hormones but also by non-hormonal factors [3]. The most commonly known role of cAMP is the transduction of signals generated by G-protein-coupled receptors to downstream cell signaling elements [4]. G-protein-coupled receptors are the largest family of membrane proteins and mediate most cellular responses to hormones and neurotransmitters [4].

G-proteins, also known as guanine nucleotide-binding proteins, are a family of proteins that act as molecular switches inside cells, and are involved in transmitting signals from a variety of stimuli from outside a cell to its interior. G-protein-coupled receptors (GPCR) constitute the largest class of cell surface receptors; GPCR genes account for 5% of the human genome [5]. Ligand binding to GPCR leads to the activation of the stimulatory subunit of the heterotrimeric G-protein, the stimulation of adenylyl cyclase, the accumulation of cAMP, the activation of cAMP-dependent protein kinase A (PKA) and the phosphorylation of proteins involved in downstream elements of the cell signaling pathway [6].

Binding of cAMP to the regulatory subunit of PKA leads to dissociation of its catalytic subunit which, in turn, phosphorylates specific serine and threonine residues on numerous target proteins, including cAMP response element (CRE) which subsequently migrates to the cell nucleus and interacts with cAMP-response element binding protein (CREB), a transcription factor involved in various intracellular processes, including proliferation, differentiation and survival. The action of CREB is regulated by cAMP-response element modulator (CREM) [7]. The field of cAMP signaling is currently witnessing exciting developments with the recognition that cAMP is compartmentalized and that the spatial regulation of cAMP is critical for faithful signal coding [8]. Hopefully, defining the organization and regulation of subcellular cAMP nanocompartments will be useful for understanding the complex functional ramifications and designing pharmacological treatments that can target GPCR and generate a blueprint that can be used to develop precision medicine interventions [8].

The cAMP subcellular nanodomains, identified in various cell types, include those located in the plasma membrane, A-kinase anchoring proteins (AKAPs) 5, 7, 9 and 12, ezrin, mitochondria (AKAP 1, AKAP 2), Ras-related protein 32, peripheral-type benzodiazepine receptor–associated protein, Wiskott–Aldrich syndrome protein verprolin homologous-1, sphingosine kinase-interacting protein, mitochondrial matrix, mitocondrial-exchange protein directly activated by cAMP, mitochondrial phosphodiesterases, Golgi apparatus and centrosome (AKAP 9, pericentrin) [8]. In spite of the fact that most of these cAMP subcellular nanodomains have not been shown to be important in gamete development and fertilization, there still remain many unresolved questions as to the mechanism of action of cAMP in the reproductive system, and these data may provide a clue to further studies into this subject in humans.

## 3. cAMP Role in Spermatogenesis

**Figure 1:**
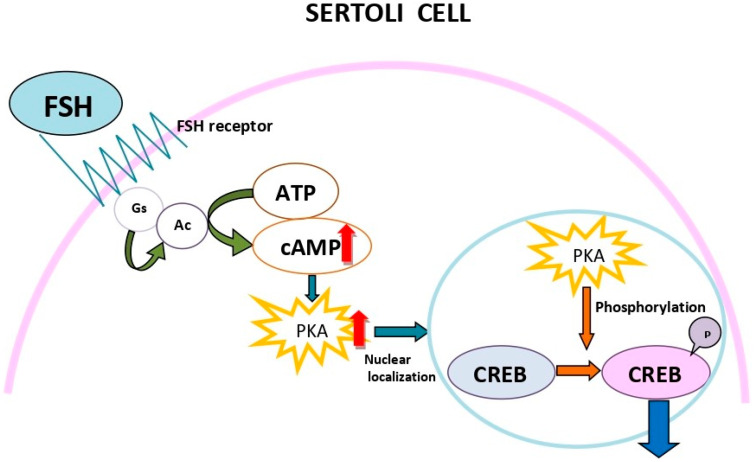
Schematic representation of cyclic adenosime monophosphate (cAMP) action in Sertoli cells. Binding of follicle-stimulating hormone (FSH) to its receptor leads to the activation of adenylyl cyclase (Ac), resulting in the synthesis of cAMP from adenosine trisphosphate (ATP). The increased level of cAMP entails the activation of protein kinase A (PKA) which, in its turn, translocates to the nucleus and activates cAMP response element binding protein (CREB) with subsequent effects on Sertoli cell support of germ cell development.

Spermatogenesis is governed by the two pituitary gonadotropins, luteinizing hormone (LH) and follicle-stimulating hormone (FSH), acting on the Leydig cells and the Sertoli cells, respectively; the former is engaged in the synthesis of testosterone (T) and the latter acts in synergy with T, responsible for the production of regulatory molecules and nutrients needed for the maintenance of spermatogenesis [9]. The fact that the action of both FSH and LH are mediated by cAMP highlights the importance of this second messenger in spermatogenesis. T activates the androgen receptor (AR) in Sertoli cells to initiate the functional responses required for spermatogenesis, especially seminiferous tubule fluid production, and reduces the expression of some androgen-dependent Sertoli cell genes [10]. As early as 2001, the CREB/CREM signaling pathway (Figure 1) was suggested to regulate spermatogenesis by regulating alternate gene expression in the testis [11]. In fact, studies in men with spermatogenic disturbance and spermatid maturation arrest demonstrated abnormal CREM expression and altered splicing events, thus strongly arguing for the essential role of CREB/CREM in sperm development in humans [11]. The same intracellular signaling pathway mediates the effect of LH in Leydig cells.

An in-vitro study performed with samples of testicular tissue, obtained by biopsies in men with obstructive azoospermia and normal spermatogenesis, showed that the two essential effects of FSH, stimulation of germ cell differentiation and inhibition of apoptosis, can be mimicked by increasing the intracellular concentration of cAMP with the use of the cAMP phosphodiesterase inhibitor pentoxifylline [12].

The essential role of CRE in cAMP signaling in the testes was confirmed in several experimental animal studies, showing that, in response to an increase in the intracellular concentration of cAMP, CREM regulates cAMP-responsive element (CRE)-mediated transcription through binding to CRE [13] and that the CREM gene generates both activators and repressors by alternative splicing in both germ and somatic cells of the testes [14,15]. The same intracellular signaling pathways are involved in the effects of LH on Leydig cells. Consequently, cAMP represents a key-regulator of sperm development (Figure 1).

## 4. cAMP Role in Oogenesis

Like spermatogenesis, cAMP is also a central, though not the only, player in mammalian oogenesis from the time point at which ovarian follicles develop an antral cavity, become responsive to the pituitary gonadotropins FSH and LH and initiate the process of oocyte maturation [16]. Oocyte maturation is a complex process consisting of various molecular regulatory events. In a simplified manner, it involves two major and largely mutually independent components, meiotic maturation and cytoplasmic maturation. In order to give rise to a developmentally competent mature oocyte, meiotic and cytoplasmic maturation have to be mutually coordinated. The completion of cytoplasmic maturation before the onset of meiotic maturation is particularly important in humans where major maternal-to-zygotic transition (MZT) of the control of early embryonic development occurs later (between the four-cell and eight-cell stage) [17,18,19] than in the mouse, the most studied animal model of early embryogenesis, where it is completed by the two-cell stage [20]. Before the onset of zygotic genome activation (ZGA), preimplantation embryo development is entirely dependent on the availability of maternal mRNA accumulation during oocyte cytoplasmic maturation, and if this process is incomplete, the embryonic development fails before ZGA can start (reviewed in [21]). 

Hence, meiotic maturation must be held back until the completion of the cytoplasmic maturation in order to allow the formation of a fully developmentally competent oocyte, and this condition is even more important in species with late MZT, such as humans, bovine animals and pigs, compared with the species with early MZT, such as rodents. cAMP signaling is intimately involved in this developmental control event. Antral follicle growth and oocyte maturation are controlled by FSH and LH, which act through the FSH receptor (FSHR) and LH receptor (LHR), respectively. As to FSHR, it displays various chemical isoforms due to both structural variations and differences in charge and functional activity [22]. Previous experiments in rats have shown that less acidic FSHR isoforms were the most active ones in intracellular cAMP release [23].

**Figure 2:**
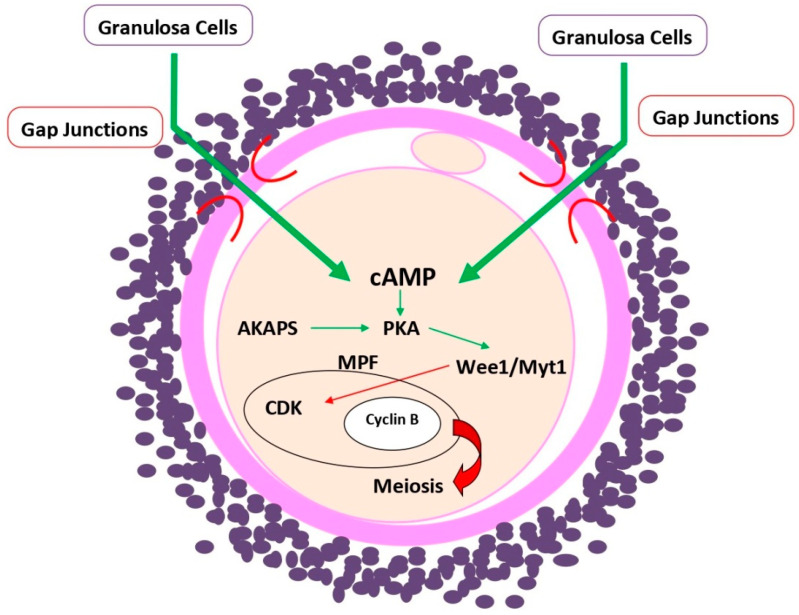
Schematic representation of cyclic adenosine monophosphate (cAMP) effect in oocytes. cAMP is mainly delivered to the oocyte from surrounding cumulus granulosa cells through their transzonal projection connecting to the oocyte via gap junctions. cAMP, together with A kinase anchoring proteins (AKAPS), make protein kinase A (PKA) capable of activating downstream protein kinases, such as Wee1/Myt/1, that inactivate the catalytic unit of maturation-promoting factor (MPF), cyclin-dependent kinase (CDK), thus maintaining meiotic arrest of the oocyte. The stimulating (green arrows) and inhibitory (red arrows) effects of the factors involved in this pathway are distinguished.

As mentioned in Section 2 of this article, the intracellular concentration of cAMP depends on an equilibrium between cAMP synthesis, by adenylyl cyclase and degradation by phosphodiesterase 3A (PDE3A). In addition to cAMP, oocyte meiotic maturation is controlled by cyclic guanosine monophosphate (cGMP), both of which are delivered to the oocyte through gap junctions from the surrounding granulosa (cumulus) cells [16]. Gap junctions are specialized intercellular connections that enable the passage of ions and small molecules, such as cAMP or cGMP, from one cell to another [24]. Although oocytes possess FSHR [25], they lack LHR [26], so the flux of cyclic nucleotides synthesized in response to these two pituitary gonadotropins is essential for the control of oocyte maturation (Figure 2). In fact, gap junctions facilitate the bidirectional flux of cyclic nucleotides between adjacent granulosa and cumulus cells and between cumulus cells adjacent to the oocyte (corona radiata cells) and the oocyte itself. Despite the physical separation of the oocyte from the innermost layer of corona radiata cells by the zona pellucida, there still remains a direct contact through transzonal projections (TZPs) of the corona radiata cells that reach the oocyte surface and mediate the nucleotide flux from the corona radiata cells to the oocyte [27].

The resumption of meiotic maturation is possible due to the active maturation promoting factor (MPF), a heterodimer consisting of two subunits: the regulatory cyclin B and the catalytic cyclin-dependent kinase (CDK) [28,29]. As the follicle grows, the continuous influx of cAMP into the oocyte maintains a high level of this second messenger in the ooplasm which, in turn, prevents the onset of oocyte meiotic maturation by maintaining a low ooplasmic level of CDK, and thus of MPF [16], acting through the phosphorylation of other types of kinases, such as Wee1/Myt1 (Figure 2). The effect of cAMP in oocytes is mediated by protein kinase A (PKA) and cAMP response element-binding protein (CREB) pathways [22]. LH activates a signal transduction pathway leading to the resumption of oocyte meiotic maturation through a breakdown of communication between the oocyte and somatic cumulus and granulosa cells, ceasing the influx of cAMP molecules into the oocyte [30]. LH surge-induced decrease in cGMP synthesis and/or increase in its hydrolysis further contribute to the lowering of cAMP levels in the oocytes through suppressing the activity of PDE3A, the enzyme responsible for cAMP degradation [16,31]. A coordinated action of cAMP and cGMP changes, in response to pituitary gonadotropins, is thus the main player in the prolonged inhibition of meiosis in oocytes as well as the resumption of meiosis once oocytes’ cytoplasmic maturation, mainly stimulated by a nongenomic effect of 17β-estradiol [32], has been achieved.

## 5. cAMP Role in Fertilization

The role of cAMP in fertilization is mainly related to changes in sperm movement and acrosome reaction, both of them closely preceding sperm entry into the oocyte. The main changes occurring in spermatozoa after ejaculation include hyperactivation, a change in sperm movement pattern required for sperm penetration of the oocyte, followed by the acrosome reaction induced by molecules present in the cumulus oophorus and the zona pellucida. cAMP is intimately involved and is indispensable for both of these key events [33]. cAMP acts through activation of PKA, as demonstrated by experiments showing that the knockout of sperm adenylyl cyclase or PKA leads to infertility due to impaired sperm motility and the inability to undergo hyperactivation [34]. PKA in cells owes its specificity and function to its localization to PKA anchoring proteins (AKAPs) [35]. Mammalian spermatozoa contain at least two AKAPs, AKAP110 and AKAP82 [33], the latter of which has been shown to mediate PKA action in the spermatozoa of both mice and humans [36]. Animal experiments have shown that perturbations of the cAMP–PKA–AKAP signaling axis disturbs sperm movement and fertilization (reviewed in [33]). Moreover, despite the fact that the main inducers of the acrosome reaction, progesterone and zona pellucida protein 3 (ZP3), do not employ cAMP in their signaling pathway, cAMP was shown to be triggered in the acrosome reaction induction in human spermatozoa downstream of the opening of store-operated calcium channels, and low levels of cAMP in spermatozoa precluded the acrosome reaction [37].

## 6. cAMP as Target of Therapeutic Action in Human Infertility

Treatments targeting cAMP to assist fertilization have been used in cases of both male and female infertility.

### 6.1. Male Infertility

In spermatozoa, the level of cAMP is tightly and dynamically regulated by two major enzymes, the adenylyl cyclases (ACs) and the phosphodiesterases (PDEs), which catalyze, respectively, the synthesis and degradation of cAMP [38]. Since the early 1990s, the PDE inhibitor pentoxifylline (PTX) has been used to increase the proportion of spermatozoa showing a hyperactivated movement pattern and to sensitize them to oocyte factors inducing the acrosome reaction, two phenomena required for the success of conventional IVF [39,40]. In spite of the fact that neither hyperactivated movement nor the acrosome reaction are required for fertilization assisted by intracytoplasmic sperm injection (ICSI), PTX is still useful for picking up living spermatozoa from samples with a total lack of sperm movement. In addition, there is a further action of PTX as a scavenger of reactive oxygen species although higher concentrations than those in current clinical use may be required to optimize this effect [40]. The observation that PTX mimics FSH’s effect on germ cell survival and differentiation in vitro [12] may justify the oral administration of PTX as an alternative means for cAMP elevation in men with high circulating FSH concentrations, leading to desensitization of the FSH receptor [41,42]. Since the use of PTX implies the risk of premature sperm acrosome reaction and thus hinders fertilization, studies are in progress to design more specific PTX analogs. Encouraging results were achieved with one particular analog, PTXm-1, which showed comparable beneficial effect on sperm motility as PTX, even at much lower concentrations, has less effect on sperm acrosome reaction, better sperm DNA integrity, extended sperm longevity and superior embryo quality [43]. In addition, other phosphodiesterase inhibitors, such as sildenafil, primarily a GMP-specific phosphodiesterase inhibitor, can increase cAMP levels in sperm cells [44]. A comparison of the usefulness of these different agents is a challenge for future research.

In addition to the data about in vitro use of PTX, there are data suggesting that in vivo oral intake of PTX can improve human sperm motility [41,45].

### 6.2. Female Infertility

Since the early 2000s, PTX has been used as an adjuvant treatment for women with thin endometria during their implantation window, in IVF attempts with both the patients’ own and donated [46,47,48] oocytes. Accumulated evidence shows a clear beneficial effect of PTX (usually combined with tocopherol) on endometrial growth and development [46,47,48]. On the other hand, it is only recently that oral treatment with PTX during ovarian stimulation was shown to result in the recovery of more good-quality mature oocytes in IVF attempts, better zygote and embryo quality, higher serum estradiol concentration and better clinical outcomes [49]. In view of the fact that the development of antral follicles and oocyte maturation are governed by FSH and LH, both acting through GPCR and the cAMP-PKA pathways, these observations can be explained by potentiating the hormone effects through the inhibition of cAMP degradation, which is particularly important in women of advanced age and/or poor ovarian reserve in whom FSH and LH receptors often fail.

## 7. Conclusions

It is well known that cAMP has functions in many tissues. The ways of manipulating the level of cAMP in patients at the level of its production are currently unknown. Consequently, the only clues for therapeutic action aimed at increasing the intracellular level of cAMP are related to the control of its biodegradation. In this article, covering the roles of cAMP in the processes of spermatogenesis, oogenesis and fertilization, the current experience with the use of PTX, the inhibitor of intracellular cAMP biodegradation, is put together. New perspectives, pointing to the development of PTX analogs with more specific actions on particular cAMP targets in germ cells, are also mentioned.

## Data Availability

Not applicable.
